# Laryngeal schwannoma treated with a CO_2_ laser: A case report

**DOI:** 10.3892/ol.2015.2858

**Published:** 2015-01-08

**Authors:** WEI KOU, CHENG ZHANG, PING WEI

**Affiliations:** 1 Department of Otolaryngology, The Children’s Hospital of Chongqing Medical University, Chongqing, P.R. China; 2 Ministry of Education Key Laboratory of Child Development and Disorders, The Children’s Hospital of Chongqing Medical University, Chongqing, P.R. China; 3 Key Laboratory of Pediatrics in Chongqing, The Children’s Hospital of Chongqing Medical University, Chongqing, P.R. China; 4 Chongqing International Science and Technology Cooperation Center for Child Development and Disorders, The Children’s Hospital of Chongqing Medical University, Chongqing, P.R. China; 5 Department of Otorhinolaryngology, The First Affiliated Hospital of Chongqing Medical University, Chongqing, P.R. China

**Keywords:** laryngeal schwannoma, CO_2_ laser, recurrence

## Abstract

Laryngeal schwannoma is a rare benign tumor of the larynx, with a high recurrence rate. The present study reports the case of a 36-year-old male patient with a two-week history of hoarseness. Electronic laryngoscopy revealed that there was a submucosal mass at the level of the right supraglottic area. A computed tomography scan of the larynx showed an 8×11-mm expansile mass in the right supraglottic area. Histopathological examination diagnosed a schwannoma of the larynx. The tumor was removed by CO_2_ laser through an endoscopic transoral approach. One year later, the patient remained symptom-free and direct examination of the larynx showed no signs of recurrence. The aim of the present study is to highlight this rare disease and its management options to the otolaryngological community.

## Introduction

Schwannomas are a type of uncommon benign nerve sheath tumor originating from the Schwann cells of the neural crest and accounting for ~5% of all head and neck tumors (1). Schwannomas always present as encapsulated, firm, slow-growing and painless masses, the majority of which are located in the parotid gland or infratemporal fossa. The tumors have also been reported in the mandible, but are rarely observed in the larynx. Symptoms rarely present themselves, but include a sore throat, difficulty in swallowing, a change of voice or a globus sensation (2–4). The use of laser applications in otorhinolaryngology has undergone significant advances over the past several years; laser technology is now used in a wide variety of procedures, and has become the primary treatment modality or standard of care for many otorhinolaryngology conditions (5). CO_2_ laser surgery is surgery using a CO_2_ laser (instead of a scalpel) to cut tissue (6). Examples include the use of a laser scalpel in otherwise conventional surgery, and soft tissue laser surgery, in which the laser beam vaporizes soft tissue with high water content (7,8). Conservative surgery has always been proposed for the treatment of this disease. The current study presents the case of a schwannoma arising in the supraglottic area in a 36-year-old male. Written informed consent was obtained from the patient.

## Case report

**Figure 1:**
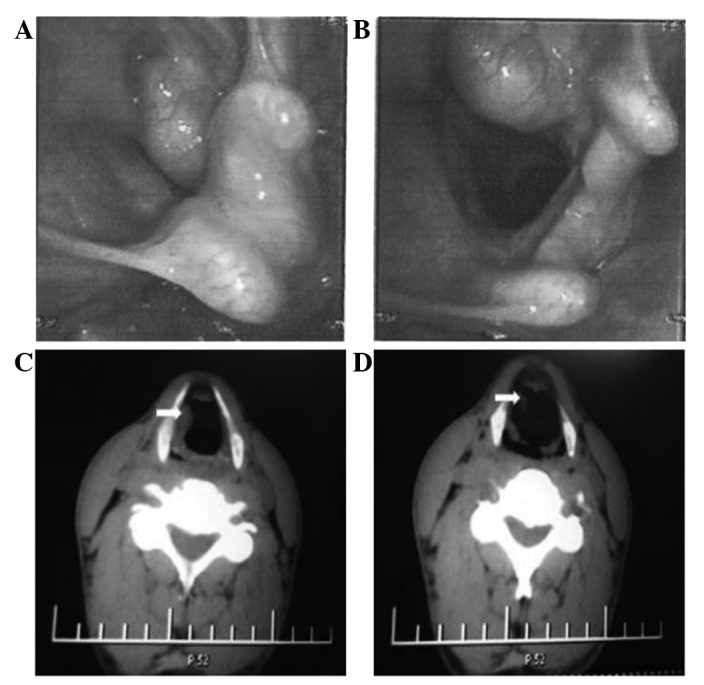
(A and B) Electronic laryngoscopy revealing a submucosal mass in the right supraglottic area; laryngeal morphology and motility were preserved. (C and D) Axial computed tomography scan showing an 8×11-mm expansile soft-tissue mass in the right supraglottic area, with no enlargement of the lymph nodes in the neck.

A 36-year-old male presented at the The First Affiliated Hospital of Chongqing Medical University (Chongqing, China) with a two-week history of hoarseness. According to the patient, other symptoms, such as a sore throat, globus sensation and difficulty in swallowing and breathing, were not present. Electronic laryngoscopy showed a submucosal mass at the level of the right supraglottic area (Fig. 1A and B). A computed tomography scan of the larynx showed an 8×11-mm expansile mass in the right supraglottic area, with well-defined boundaries. There was no enlargement of the lymph nodes in the neck (Fig. 1C and D). The differential diagnosis of the tumor included squamous cell carcinoma, fibroma, neurofibroma, lymphoma and schwannoma.

**Figure 2:**
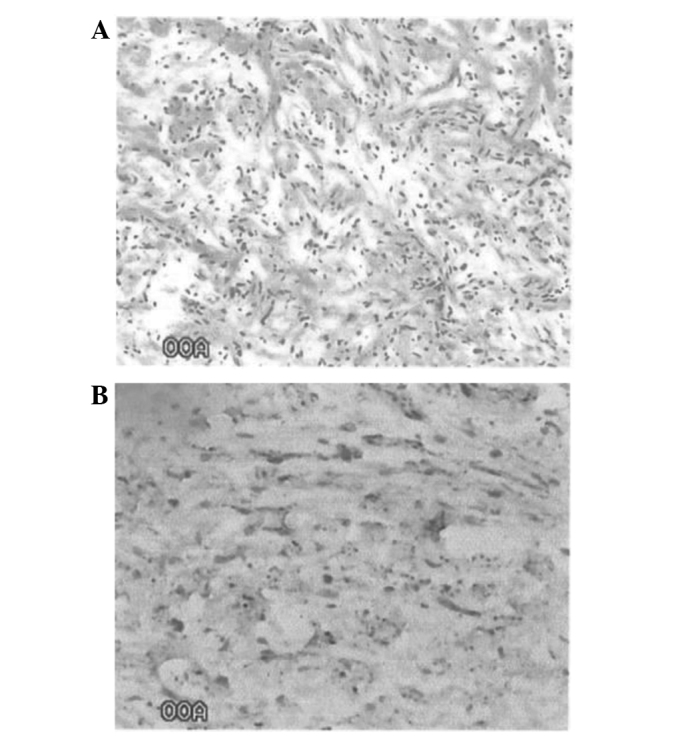
Histopathological images. (A) Benign spindle cell lesion (hematoxylin and eosin stain; magnification, ×20). (B) Schwannoma of the larynx, with diffuse positivity for S-100 (magnification, ×40).

**Figure 3:**
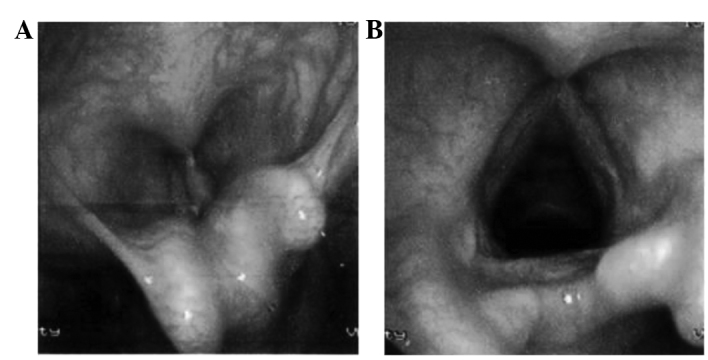
(A and B) Electronic laryngoscopy revealing complete healing of the larynx, without recurrence, one year after surgery. Laryngeal morphology and motility were preserved.

Resection of the tumor was performed by CO_2_ laser through an endoscopic transoral approach under general anesthesia. During the surgery, the tumor was completely resected along the tumor boundary. No nerves were connected caudally to the tumor. Histopathological examination of the frozen section demonstrated that the tumor was mainly composed of active spindle cells. Next, ~2 mm of tissue was resected along the edge of the tumor by CO_2_ laser. Histopathological examination subsequent to the surgery demonstrated benign spindle cell lesions (Fig. 2A). Immunohistochemically, the tumor was strongly positive for cluster of differentiation 56 and S-100 (Fig. 2B), but negative for smooth muscle actin, vimentin and maltose-binding protein. On the basis of these findings, the tumor was diagnosed as a schwannoma of the larynx, originating from the distal portion of the internal branch of the superior laryngeal nerve or the recurrent laryngeal nerve. The patient has since been followed up for 12 months and no evidence of recurrence has been observed (Fig. 3A and B).

## Discussion

Schwannomas are slow-growing, benign tumors of the nerve sheath, first described by Verocay in 1908 (9). Since then, few cases have been reported in the literature (10). A study by Enzinger and Weiss (11) suggested that the histological diagnosis of schwannoma could be characterized by three features: i) Encapsulation; ii) the presence of Antoni A and B areas; and iii) a positive reaction for S-100. The present case exhibited each of these features, and the tumor was well-defined; these findings eventually led to a diagnosis of schwannoma.

The majority of laryngeal schwannomas occur in the supraglottic area and are present at any age. The etiology of schwannomas is not well understood. Surgical excision is the main strategy for treatment. Furthermore, the preservation of laryngeal function and protection of the laryngeal mucosa from injury during surgery must also be guaranteed. However, recurrences have been reported by long-term follow-up subsequent to conventional surgery (2).

CO_2_ lasers are widely used to remove thin layers from the surface of the mucosa without undermining the deeper layers. This procedure can be performed with fairly little bleeding, swelling, pain or scarring. The lasers are more precise than scalpels and the high temperature generated by the lasers aids in cleaning the edges of the body tissue that it is cutting, reducing the risk of infection and recurrence. Using this approach, the surgery time may be reduced and the healing time may be shortened. CO_2_ lasers now play an increasingly important role as a minimally invasive alternative to conventional surgical interventions for patients in a number of oncology services (12).

Although differing surgical approaches for removing laryngeal schwannomas have been discussed in the literature, including endoscopic removal and a laryngofissure approach (13,14). The use of a CO_2_ laser via a transoral approach can be applied in patients with early-stage laryngeal carcinoma and atlanto-axial vertebral chronic dislocation (15) In the present case, the tumor was resected by CO_2_ laser, demonstrating that safe removal of the tumor is possible using this approach, without severe injury to the laryngeal mucosa and with low recurrence.
